# 新型非实性肺小结节恶性概率预测模型的构建与验证

**DOI:** 10.3779/j.issn.1009-3419.2019.01.06

**Published:** 2019-01-20

**Authors:** 飞 肖, 其多 余, 真榕 张, 德若 刘, 朝阳 梁

**Affiliations:** 100029 北京，中日友好医院胸外科 Department of Thoracic Surgery, China-Japan Friendship Hospital, Beijing 100029, China

**Keywords:** 肺小结节, 肺肿瘤, 预测模型, 恶性概率, Pulmonary nodule, Lung neoplasms, Predictive model, Probability of malignancy

## Abstract

**背景与目的:**

数学预测模型是判断肺小结节恶性概率的有效工具。伴随肺癌流行病学趋势的改变，以非实性肺小结节为影像学表现的早期肺癌检出率逐年升高，准确鉴别并及时治疗干预可有效改善预后。本研究旨在专门针对非实性肺小结节构建新型恶性概率预测模型，为有创诊疗提供客观依据，并尽量避免不必要的侵袭性操作及其可能造成的严重后果。

**方法:**

回顾性分析自2013年1月-2018年4月，单中心经穿刺活检或手术切除获得明确病理诊断的362例非实性肺小结节病例资料，包括临床基本资料、血清肿瘤标记物和影像学特征等。病例分两组，应用建模组数据做单因素分析和二分类*Logistic*回归，判定独立危险因素，建立预测模型；应用验证组数据验证模型预测价值并与其他模型比较。

**结果:**

362例非实性肺小结节病例中，313例（86.5%）确诊为非典型腺瘤样增生（atypical adenomatous hyperplasia, AAH）/原位腺癌（adenocarcinoma *in situ*, AIS）、微浸润腺癌（minimally invasive adenocarcinoma, MIA）或浸润性腺癌，49例诊断为良性病变。年龄、血清肿瘤标记物癌胚抗原（carcino-embryonic antigen, CEA）和Cyfra21-1、肿瘤实性成分比值（consolidation tumor ratio, CTR）、分叶征和钙化被确定为独立危险因素。模型受试者工作曲线下面积为0.894。预测灵敏度为87.6%，特异度为69.7%，阳性预测94.8%，阴性预测值为46.9%。经验证模型预测价值显著优于VA、Brock和GMUFH模型。

**结论:**

本研究建立的新型非实性肺小结节恶性概率预测模型具备较高的诊断灵敏度和阳性预测值。经初步验证，其预测价值优于传统模型。未来经大样本验证后，可用作高危非实性肺小结节活检或手术切除前的初筛方法，具备临床应用价值。

## Abstract (en)

**Background and objective:**

Mathematical predictive model is an effective method for preliminarily identifying the malignant pulmonary nodules. As the epidemiological trend of lung cancer changes, the detection rate of ground-glass-opacity (GGO) like early stage lung cancer is increasing rapidly, timely and proper clinical management can effectively improve the patients' prognosis. Our study aims to establish a novel predictive model of malignancy for non-solid pulmonary nodules, which would provide an objective evidence for invasive procedure and avoid unnecessary operation and the consequences.

**Methods:**

We retrospectively analyzed the basic demographics, serum tumor markers and imaging features of 362 cases of non-solid pulmonary nodule from January 2013 to April 2018. All nodules received biopsy or surgical resection, and got pathological diagnosis. Cases were randomly divided into two groups. The modeling group was used for univariate analysis and logistic regression to determine independent risk factors and establish the predictive model. Data of the validation group was used to validate the predictive value and make a comparison with other models.

**Results:**

Of the 362 cases with non-solid pulmonary nodule, 313 (86.5%) cases were diagnosed as AAH/AIS, MIA or invasive adenocarcinoma, 49 cases were diagnosed as benign lesions. Age, serum tumor markers CEA and Cyfra21-1, consolidation tumor ratio value, lobulation and calcification were identified as independent risk factors. The AUC value of the ROC curve was 0.894, the predictive sensitivity and specificity were 87.6%, 69.7%, the positive and negative predictive value were 94.8%, 46.9%. The validated predictive value is significantly better than that of the VA, Brock and GMUFH models.

**Conclusion:**

Proved with high predictive sensitivity and positive predictive value, this novel model could help enable preliminarily screening of "high-risk" non-solid pulmonary nodules before biopsy or surgical excision, and minimize unnecessary invasive procedure. This model achieved preferable predictive value, might have great potential for clinical application.

依据2017版Fleischner协会指南，肺结节按影像学特征分为实性结节和非实性结节，后者又依据毛玻璃密度成分（ground glass opacity, GGO）比例分为部分实性结节（part-solid nodule, PSN）和纯磨玻璃样结节（pure ground glass nodule, PGGN）^[1]^。伴随低剂量螺旋CT在肺癌筛查中的普及和高分辨率计算机断层扫描（computed tomography, CT）的广泛应用，肺小结节的检出率逐年升高。美国国家肺癌筛查研究（National Lung Screening Trial, NLST）已经证实低剂量CT筛查可降低肺癌相关病死率^[2, 3]^。目前，以美国国家综合癌症网络（National Comprehensive Cancer Network, NCCN）指南为代表^[4]^，多项指南对肺结节的检出方法、随访策略、手术适应证等做出详细规定。其中，临床经验诊断高危组对象应接受包括CT引导下穿刺或腔镜活检在内的有创检查。尽管美国胸科医师协会（American College of Chest Physicians, ACCP）按照年龄、吸烟史和病变大小、位置、形态等对“危险程度”有粗略的分组^[5]^，目前仍缺乏对“高危”的统一、确切定义。尤其是随着肺癌流行病学趋势的改变，在亚洲地区，以非实性肺小结节为表征的早期肺癌在年轻不吸烟女性患者中同样高发^[6-8]^，影响肺癌筛查、诊疗策略的制定与选择。

因此，有必要建立新的预测模型，针对非实性肺小结节，应用数学计算方法给出客观的“恶性概率”。与以往报道的模型不同，本项研究的对象是临床怀疑恶性的非实性肺小结节，致力于在临床肺活检或手术切除前进行更准确判断，提高模型预测灵敏度，为有创操作提供更客观的依据，同时提高模型的阳性预测价值，尽量避免不必要的侵袭性操作，降低肺癌筛查相关副损伤，减少不必要的医疗支出和资源浪费。考虑到预测模型仅仅用于初筛的定位，且正电子发射断层显像（positron emission tomography-computed tomography, PET-CT）检查对肺小结节，尤其是直径小于1 cm的肺结节诊断价值存在争议^[9]^，更无论在一些低收入地区，其性价比仍是客观考虑因素之一，本研究未纳入PET-CT相关检查指标。同时，对一些技术水平要求较高的新型诊疗技术方法，如“CT三维成像”等，为了便于临床应用，增加模型的适用性，亦暂未纳入。

## 1 材料与方法

### 1.1 入组标准及排除标准

参考NCCN指南中肺小结节诊疗策略在近年来的变化^[4, 10]^，我们将初始直径≥5 mm且 < 1 cm，随访胸部CT提示病变体积增大或实性成分比例增加，或者直径≥1 cm胸部CT高度怀疑恶性作为非实性肺小结节的穿刺或手术切除活检标准，这同时也是本项研究中病例的入组标准。排除标准包括病变实性成分最大径 > 3 cm，既往5年内有肺恶性肿瘤史，以及同时多发肺小结节病例。本项回顾性临床研究经中日友好医院临床试验伦理委员会审查通过。入组患者接受有创检查或手术前已签署相关知情同意书。

### 1.2 病例队列

2013年1月-2018年4月，经初筛共398例患者满足上述入组条件，纳入回顾研究。全部患者经穿刺活检或手术切除获得明确病理诊断，其中36例因临床资料不完备予以剔除，余362例构成研究队列（详细情况见表 1）。病例按总体良恶性比例随机分为两组，其中建模组242例，约占2/3，验证组120例。收集患者年龄、性别、吸烟史（包括是否戒烟、戒烟时间等）、家族肺癌病史和肺外肿瘤史等基本资料。记录穿刺或手术活检前7天内外周血肿瘤标记物CEA和Cyfra21-1水平。回顾性阅片，判定肺结节的大小（按照Fleischner协会指南肺结节测量方法^[1]^和非小细胞肺癌（non-small cell lung cancer, NSCLC）第8版TNM分期中T分期标准^[11]^，分别测量结节整体和实性部分的长轴）并计算肿瘤实性成分比值（consolidation tumor ratio, CTR），记录病变位置和形态（包括毛刺状、分叶征、钙化、空泡、边界清楚、胸膜牵拉征和合并肺气肿等）。

**1:** 入组患者一般状况、血清肿瘤标记物水平及影像学特征 Patients'characteristics and demographic data

Characteristics	*n*=362
Demographic data	
Age (Mean±SD, yr)	55.2±11.1
Male (%)	48.3
Smoking history (%)	27.6
Family history of lung cancer (%)	18.8
History of malignancy (%)	4.1
Serum tumor markers	
CEA (Mean±SD, ng/mL)	2.99±1.77
Cyfra21-1 (Mean±SD, ng/mL)	2.49±0.93
Imaging characteristics	
Maximum diameter (Mean±SD, cm)	1.76±0.72
Diameter of the solid component (Mean±SD, cm)	0.63±0.56
CTR≥50% (%)	36.7
Located on upper lobe (%)	56.6
Spiculation (%)	34.0
Lobulation (%)	63.3
Calciﬁcation (%)	1.4
Cavitation (%)	5.2
Clear margin (%)	64.9
Pleural retraction sign (%)	17.1
Emphysema (%)	7.5
SD: standard deviation; CEA: carcino-embryonic antigen; CTR: consolidation tumor ratio.	

### 1.3 统计学分析

应用SPSS 19.0软件进行统计分析。将年龄、性别、吸烟史、家族肺癌病史、肺外肿瘤史、CEA、Cyfra21-1、结节最大径、实性成分最大径、CTR值、位于上叶、毛刺状、分叶征、钙化、空泡、边界清楚、胸膜牵拉及合并肺气肿等18项作为潜在危险因素，先行单变量分析，其中连续变量应用*t*检验，分类变量应用χ^2^检验。将单因素分析差异有统计学意义的变量（为避免漏掉重要因素，*P*值上限放宽至0.25）纳入二分类*Logistic*回归模型，得到判定非实性肺小结节恶性概率的独立危险因素，计算比值比（odd ratio, OR）及95%置信区间（confidence interval, CI）。基于多变量分析结果建立预测模型，计算“约登指数（Youden Index）”确定模型诊断界值，得到模型诊断的灵敏度、特异性、阳性预测值和阴性预测值。绘制受试者工作曲线（receiver-operating characteristic curve, ROC curve），应用MedCalc 12.5软件，基于验证组数据，通过比较ROC曲线下面积（area under the curve, AUC），与现有各肺小结节恶性概率预测模型进行验证、比较，*P* < 0.05为有统计学差异。

## 2 结果

### 2.1 病理诊断结果

362例非实性肺小结节病例中，313例（86.5%）诊断为恶性病变，其中CT引导下穿刺活检明确恶性52例，进一步手术切除后，共病理确诊AAH/AIS 16例，MIA 52例，浸润性腺癌245例；49例诊断为良性病变，其中CT引导下穿刺未见恶性成分6例（均建议继续定期随访，必要时反复穿刺甚至手术切除明确病理诊断），手术切除43例中，诊断机化性肺炎或其他炎性病变37例，肺内淋巴结4例，隐球菌感染2例。

### 2.2 恶性概率独立危险因素分析及预测模型构建

按照良恶性分组，比较组间基本临床资料、血清肿瘤标记物水平和影像学特征差异情况；同时，如前述按照总体良恶性比例随机选取242例病例构成建模组，其中恶性肺小结节病例209例，良性33例，对上述18项潜在危险因素行单变量分析，结果如表 2示。

**2:** 非实性肺小结节良恶性组间单因素分析结果 Demographic data, serum tumor markers and imaging characteristics of the non-solid lung nodules in benign and malignant groups

Characteristics	Study cohort (*n*=362)			Derivation set (*n*=242)		
	Benign (*n*=49)	Malignant (*n*=313)	*P*	Benign (*n*=33)	Malignant (*n*=209)	*P*
Demographic data						
Age (Mean±SD, yr)	47.8±7.3	56.4±11.2	< 0.001	48.0±7.9	56.9±11.3	< 0.001
Male (%)	42.9 (21/49)	49.2 (154/313)	0.409	48.5 (16/33)	50.2 (105/209)	0.851
Smoking history (%)	24.5 (12/49)	28.1 (88/313)	0.598	27.3 (9/33)	28.7 (60/209)	0.865
Family history of lung cancer (%)	16.3 (8/49)	19.2 (60/313)	0.636	15.2 (5/33)	19.6 (41/209)	0.543
History of malignancy (%)	2.0 (1/49)	4.5 (14/313)	0.683	0 (0/33)	4.3 (9/209)	0.472
Serum tumor markers						
CEA (Mean±SD, ng/mL)	1.66±0.81	3.20±1.79	< 0.001	1.72±0.73	3.20±1.80	< 0.001
Cyfra21-1 (Mean±SD, ng/mL)	1.94±0.69	2.58±0.94	< 0.001	1.77±0.67	2.53±0.89	< 0.001
Imaging characteristics						
Maximum diameter (Mean±SD, cm)	1.66±0.62	1.77±0.74	0.293	1.64±0.63	1.70±0.74	0.641
Diameter of the solid component (Mean±SD, cm)	0.33±0.36	0.67±0.57	< 0.001	0.29±0.35	0.64±0.58	< 0.001
CTR≥50% (%)	20.4 (10/49)	39.3 (123/313)	0.011	18.2 (6/33)	40.2 (84/209)	0.015
Located on upper lobe (%)	55.1 (27/49)	56.9 (178/313)	0.816	60.6 (20/33)	57.9 (121/209)	0.769
Spiculation (%)	20.4 (10/49)	36.1 (113/313)	0.031	24.2 (8/33)	35.4 (74/209)	0.208
Lobulation (%)	34.7 (17/49)	67.7 (212/313)	< 0.001	36.4 (12/33)	62.7 (131/209)	0.004
Calciﬁcation (%)	6.1 (3/49)	0.6 (2/313)	0.019^*^	6.1 (2/33)	0.5 (1/209)	0.050^*^
Cavitation (%)	4.1 (2/49)	5.4 (17/313)	0.961	6.1 (2/33)	5.7 (12/209)	1.000
Clear margin (%)	75.5 (37/49)	63.3 (198/313)	0.095	75.8 (25/33)	64.1 (134/209)	0.190
Pleural retraction sign (%)	6.1 (3/49)	18.8 (59/313)	0.028	9.1 (3/33)	21.1 (44/209)	0.106
Emphysema (%)	10.2 (5/49)	7.0 (22/313)	0.621	12.1 (4/33)	8.6 (18/209)	0.745
^*^Applying *Fisher* exact method.						

总体研究队列中，良恶性患者组间在年龄、血清CEA和Cyfra21-1水平、结节实性成分最大径、CTR值、影像学特点毛刺状、分叶征、钙化和胸膜牵拉等方面存在显著差异。良性结节更多表现为边界清楚，但未及显著差异。良恶性患者组间在性别比例、吸烟史、家族肺癌病史、肺外肿瘤史、结节最大径、位置、空泡征和合并肺气肿比例上无差异。建模组队列中，良恶性患者组间在年龄、血清CEA和Cyfra21-1水平、结节实性成分最大径、CTR值、影像学特点分叶征和钙化等方面同样存在显著差异。综合以上结果，将年龄、CEA、Cyfra21-1、实性成分最大径、CTR值、毛刺状、分叶征、钙化、边界清楚及胸膜牵拉等10项作为潜在危险因素（*P* < 0.25），纳入多因素分析，应用二分类*Logistic*回归模型，得到判定非实性肺小结节恶性概率的独立危险因素，及其OR值及95%CI。结果如表 3示。

**3:** 非实性肺小结节恶性概率独立危险因素多因素分析结果 Multivariate analysis of independent predictors of malignancy for non-solid lung nodules

	Regression coefficient	*P*	OR	95%CI	
				Lower	Upper
Age (yr)	0.055	0.023	1.056	1.008	1.107
CEA (ng/mL)	1.126	< 0.001	3.082	1.699	5.590
Cyfra21-1 (ng/mL)	0.877	0.014	2.404	1.194	4.839
CTR≥50%	1.337	0.021	3.807	1.219	11.885
Lobulation	0.980	0.047	2.665	1.013	7.012
Calciﬁcation	-3.236.	0.021	0.039	0.003	0.610
OR: odd ratio; CI: confidence interval.					

患者年龄、血清学肿瘤标记物CEA和Cyfra21-1、CTR值、分叶征和钙化被确定为非实性肺小结节恶性概率独立危险因素。实性成分最大径（*P*=0.925）、毛刺状（*P*=0.983）、边界清楚（*P*=0.968）和胸膜牵拉（*P*=0.473）为非独立危险因素。

据此，提出中日医院非实性肺小结节恶性概率预测模型（China-Japan Friendship Hospital model, CJFH model）：恶性概率（probability of malignancy）= eX/（1+eX）。其中，X = -6.159+（0.055×年龄）+（1.337×CTR）+（0.980×分叶征）-（3.236×钙化）+（1.126×CEA）+（0.877×Cyfra21-1）。

数字e是一个数学常数，是自然对数函数的底数。年龄按年/岁记录；CTR如果大于或等于0.5记为1，小于0.5记为0；分叶征和钙化若存在记为1，否则为0；CEA和Cyfra21-1代表外周血肿瘤标记物CEA和Cyfra21-1水平，单位为ng/mL。

基于建模组数据构建CJFH模型的ROC曲线（图 1），得到AUC值为0.894（95%CI: 0.846-0.942）。为尽量避免漏诊恶性结节，并减少不必要的手术或活检，结合约登指数计算结果，确定为0.794诊断阈值，此时模型灵敏度为87.6%（183/209），特异度为69.7%（23/33），阳性预测值为94.8%（183/193），阴性预测值为46.9%（23/49）。

**1:**
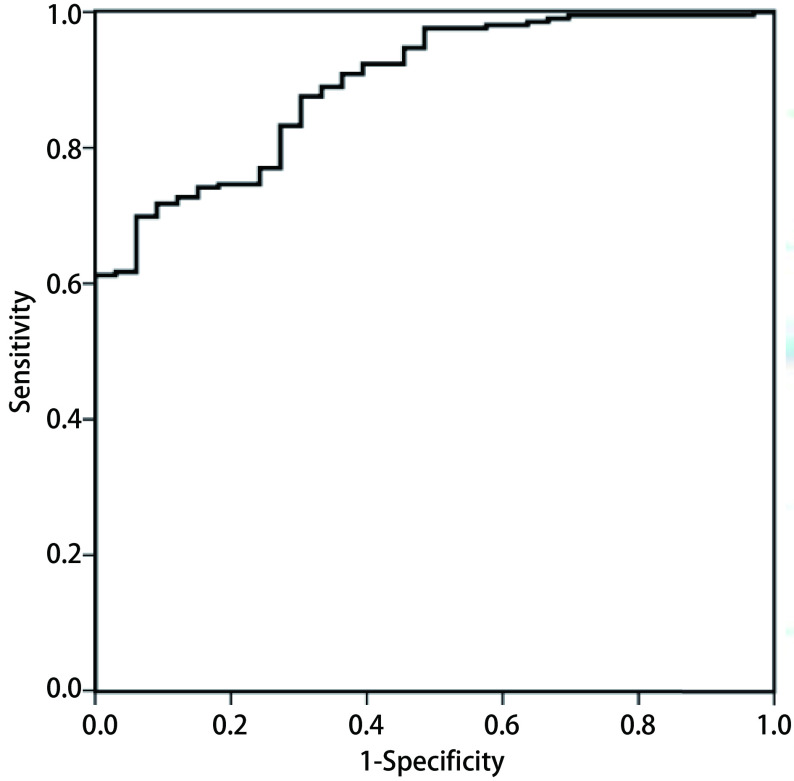
基于建模组数据绘制CJFH模型ROC曲线 ROC curve of the CJFH model based on data from the derivation set

### 2.3 对恶性概率预测模型的验证和比较

应用验证组120例病例数据，代入CJFH模型和其他广泛应用的肺小结节恶性概率预测模型，包括Mayo模型^[12]^、VA模型^[13]^、Brock模型^[14]^、北京大学人民医院（Peking University People’s Hospital, PKUPH）模型^[15]^和广州医科大学第一医院（Guangzhou Medical University First Hospital, GMUFH）模型^[16]^（各模型具体计算公式见附录），绘制各自模型的ROC曲线（图 2），得到AUC值。CJFH模型ROC曲线的AUC值与其他模型ROC曲线的AUC值比较结果如表 4示。

**2:**
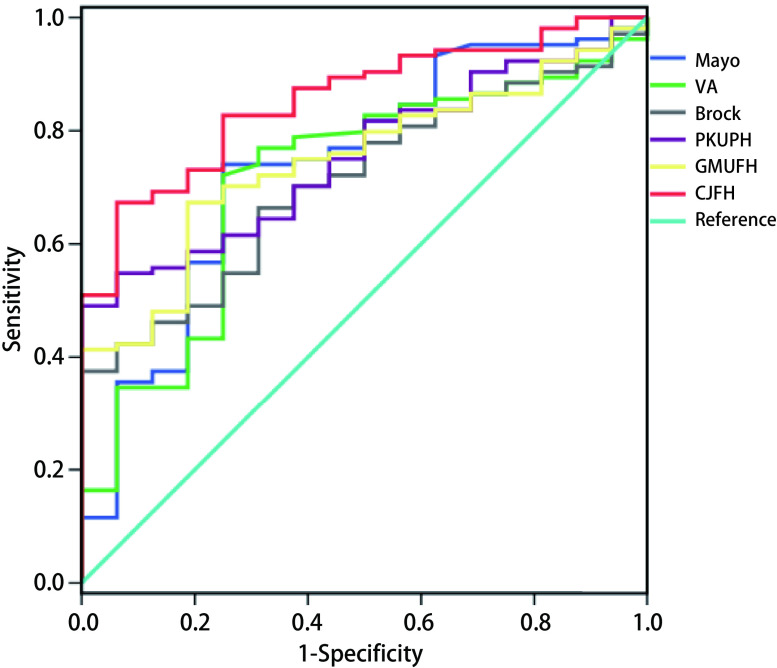
基于验证组数据绘制不同预测模型的ROC曲线 ROC curves of different models based on data from the validation set

**4:** 基于验证组数据比较CJFH模型与现有模型的AUC值 Comparison result of AUC values between CJFH model and other models

Models	AUC	*P*	95%CI	
			Lower	Upper
CJFH model	0.855	-	0.775	0.934
Mayo model	0.739	0.065	0.606	0.871
VA model	0.715	0.031	0.585	0.844
Brock model	0.709	0.020	0.598	0.820
PKUPH model	0.755	0.077	0.656	0.853
GMUFH model	0.748	0.035	0.644	0.851
AUC: area under the curve; CJFH: China-Japan Friendship Hospital; PKUPH: Peking University People’s Hospital; GMUFH: Guangzhou Medical University First Hospital.				

基于验证组数据，得到CJFH模型ROC曲线AUC值为0.855（95%CI: 0.775-0.934），提示预测效果较为满意。该AUC值显著高于VA、Brock、GMUFH模型ROC曲线的AUC值（*P* < 0.05），亦有优于Mayo模型和PKUPH模型的趋势，但未及显著差异。

## 3 讨论

建立肺小结节恶性概率预测模型，有助于为进一步有创诊断、治疗提供“客观”依据，是对肺小结节诊治流程的有益补充。自从Swensen等建立了Mayo模型^[12]^后，已有较多肺小结节恶性概率预测模型^[13-16]^相继问世。受入组病例构成的影响，传统预测模型更多针对实性肺小结节，仅有Brock模型^[14]^依据结节类型做了系数调整。已知实性肺小结节和以GGO为主要影像学表现的非实性肺小结节相比，其临床、病理特征均有较大差别^[17-19]^。近年来，尤其在亚洲地区，以非实性肺小结节为表征的早期肺癌检出率逐年升高，且患者群体中大量为年轻不吸烟女性。肺癌流行病学趋势的改变提示有必要为非实性肺小结节构建独立的恶性概率预测模型。

本研究回顾性分析了单中心362例患者的临床资料。全部患者临床考虑“高危”，均接受了穿刺活检或手术切除取得明确病理诊断。总体上看，86.5%（313/362）的患者确诊为肺恶性肿瘤，良恶性患者组间在性别比例、吸烟史、家族肺癌病史、肺外肿瘤史、结节最大径、位置、空泡征和合并肺气肿比例上无差异，这反映出以非实性肺小结节为表征的肺恶性肿瘤有别于传统肺癌，具备不同的临床病理特征。例如，女性患者比例增加；不吸烟并不能作为保护性因素；病变位置不易区别良恶性；尤其是病灶直径——整体的大小并不能影响恶性概率，亦与肺癌分期及预后无关^[20]^，这在一定程度上符合NSCLC第8版TNM分期中对T分期的修订^[11]^。本研究确认年龄、血清学肿瘤标记物CEA和Cyfra21-1、CTR值、分叶征和钙化为非实性肺小结节恶性概率独立危险因素，并纳入模型。调整年龄所占权重，并增加血清肿瘤标记物水平作为预测值，这符合既往研究中提出的改善建议^[21]^。分叶征和钙化被证实为影像学特征上影响恶性概率的独立危险因素，既往亦有报道^[22]^。针对非实性肺小结节的特点，引入CTR作为预测值，与文献报道应用CTR区分肿瘤侵袭性和安排随访相似^[17]^。Takenaka等^[23]^报道病灶实性成分体积是临床Ia期NSCLC无病生存期的独立危险因素，但在本研究多因素分析中，实性成分最大径并不是非实性肺小结节恶性概率的独立危险因素。

本模型具备较高的预测价值，ROC曲线AUC值为0.894。当以0.794为诊断阈值时，模型灵敏度达87.6%，阳性预测值达94.8%。高灵敏度和阳性预测值可以保证预测模型用作活检或外科切除前的初筛方法，具备临床应用价值，最大可能的避免漏诊肺癌病例，同时尽量降低诊断假阳性率，避免不必要的活检、手术对患者造成副损伤，减少医疗费用支出和资源浪费。模型的特异度和阴性预测值较低，分别为69.7%和46.9%，一方面，这与本预测模型用作活检或外科切除前初筛的定位及其使用目的有关；另一方面，说明应用模型预测后得到一定数量假阴性（26/242）和假阳性（10/242）结果，其中假阴性更多，提示对推测良性可能性大的非实性结节同样应当注重随访，依据动态变化情况选择进一步治疗策略。和其他模型相比，本模型的灵敏度和阳性预测值大幅占优，但特异度和阴性预测值与国外模型接近，低于国内模型的有关报道（PKUPH模型：特异度81.8%，阴性预测值85.7%；GMUFH模型：特异度84.6%，阴性预测值83.0%）。

从总体预测价值的角度看，经验证组队列验证，与其他模型相比，本模型预测价值占优，且显著优于VA模型、Brock模型和GMUFH模型。可能有如下几方面原因：(1)研究对象确定为临床可疑恶性的非实性肺小结节，针对特定人群，避免了病例选择偏倚；(2)病例队列来自国人群体，亚裔肺癌患者中不吸烟、女性比例更高^[6, 7]^，且亚裔罹患结核的比例更高，可能对病灶形态及位置分布产生影响；(3)模型引入了新型预测值，包括反映非实性肺小结节影像学特征的CTR值，和血清肿瘤标记物水平。尤其是后者，尽管肺癌血清肿瘤标记物的敏感性和特异性普遍不高，有研究证实CEA和Cyfra21-1水平对早期肺癌诊断有一定价值，并可能对其预后产生影响^[24, 25]^。此外，就各个模型自身的局限性逐一分析：Mayo模型作为20多年前的研究成果，存在地域和种族的局限性，研究还除外了5年内罹患肺癌或有肺外肿瘤病史者，削弱了样本的代表性。此外，统计结果中的恶性肺小结节所占比例较低，更有12%的患者无明确的病理诊断，仅根据2年随访结节无变化即定为良性，考虑到大量以GGO为表象的早期肺癌可连续随访多年而没有变化，其统计方式有待推敲和完善。VA模型提出影响肺小结节恶性概率的独立危险因素包括年龄、吸烟史、戒烟时间和结节直径。相较于其他模型，该模型并未包含对结节影像特征的评价因素，可能导致较大的误差。Brock模型是目前收集样本例数最多、国际公认诊断效能最强的预测模型之一，但其建模数据源于加拿大一项肺癌筛查实验的病例队列，入组的均为首次经CT筛查发现的肺小结节。当使用Brock模型用于“高危”非实性肺小结节患者穿刺或手术前粗筛时，存在一定程度的选择偏倚。国内相关模型同样面临类似的选择偏倚问题，此外，尚无预测模型将血清肿瘤标记物水平纳入，这可能是造成模型预测价值较低的瓶颈因素。

本研究同样存在局限。作为单中心回顾性研究，病例数量有限，结果尚需大样本病例验证。本模型临床应用定位于初筛，为兼顾实用性和准确性，并未纳入一些客观条件要求较高、尚未广泛普及的变量。注意到近年来新报道的肺小结节预测模型中，有将PET-CT检查标准摄取值（standard uptake value, SUV）^[26]^或血浆microRNA水平^[27]^纳入预测模型获得较高诊断效能的实例，这些探索性的研究结果尚待大样本病例队列加以验证。在本项研究的设计过程中，考虑到数据采集的可操作性和质控要求，并未纳入术前检查容易缺漏或“推测”与恶性概率无关的指标。但确有研究证实如血清总蛋白水平、实测1秒量占预计值百分比等同样可以是肺小结节恶性概率的预测指标^[28]^。提示前瞻性的实验设计和更加完备的建模方法是今后研究的方向。

综上，本研究在兼顾实用性和准确性的前提下，建立了新型非实性肺小结节恶性概率预测模型，预测指标包括年龄、血清学肿瘤标记物CEA和Cyfra21-1、CTR值、分叶征和钙化。模型具备理想的预测价值，具备较高的诊断灵敏度和阳性预测值。经初步验证，对非实性肺小结节恶性概率的预测价值优于传统模型。未来经大样本病例验证后，可用作高危非实性肺小结节活检或手术切除前的初筛方法，具备临床应用价值。
